# Remarks on Pleurodynia

**Published:** 1854-05

**Authors:** Wm. J. Chenoweth

**Affiliations:** Darwin, Illinois


### ART. III.—REMARKS ON PLEURODYNIA.

BY WM. J. CHENOWETH, M. D., OF DARWIN, ILLINOIS.

I have observed in quite a number of cases of intermittent fever, that Pleurodynia accompanied them. In nearly every case that I have been called on to treat the impression of the friends or parents was that the patient had pleurisy or pneumonia, and they were rather incredulous when told that there was nothing serious the matter. I was more forcibly reminded of this form of Pleurodynia by a Homœopathist who insisted that he could cure every case of pneumonia he met, in two or three days, and detailed the case of a blacksmith who was attacked suddenly and violently with pain in his side increased by taking a long breath and who was relieved in less than twenty-four hours so as to be able to go to work. I suggested that it might have been neuropathic, but although he admitted that he did not make a physical exploration of the chest with the view of determining, yet he was perfectly satisfied of the nature of the affection; he had seen it often, and had been in the habit of treating it for years.

In Kentucky I had often seen pleurodynia, but I had never seen it assume an intermittent form, and when I first met the disease here I was surprised to find that it did not yield to rubefacients and rest, but seeing it assume a periodic form I treated it successfully with quinine, and the disease being of frequent occurrence and always periodic I was finally led to look upon it as one of the protean forms of miasmatic fever.

On the 9th day of Dec., 1853, I saw a boy six years old who had suffered for about two months with an intermittent fever, but was now convalescent. He was taken, a few hours before I saw him, with severe pain under his right nipple which extended to the scapula of that side. He would cry out at every inspiration. I examined him as carefully as I could but did not detect the least abnormal sound in his respiration, the vesicular murmur was clear and distinct. I ordered him to be rubbed over the affected side with volatile liniment. The next evening he was suffering as on the previous day, having been attacked about the same hour. I now gave him quinine and ordered the liniment to be rubbed over the spine. I heard no more of the pain for about two weeks, when he was again attacked as before, and this time it yielded at once to the same remedies I had before employed.

In February, 1854, a Mr. Adams called at my office to be cured of a quartian ague accompanied with a distressing pain in his left side and under the scapula of the same side; I rubbed a liniment composed of spirits of turpentine and chloroform over the spine, which shortened the paroxysm more than one half. I ordered him to have his spine rubbed at the next expected time of the chill; he did as directed and had neither chill nor pain. But it again returned in a few days and was again cured in the same manner.

In the early part of March I went three miles to see a boy eight years old, said to have pleurisy. I found him suffering with an intermittent pleurodynia which yielded readily to quinine.

I was called on to prescribe for a boy twelve years old, in town, on the 12th of April; the boy's father had been at my house several times during the afternoon, and was very impatient to see me, but having gone to the country I did not return until after dark. I went to see him as soon as I returned, and found him in great distress, scarcely able to draw a breath without crying, and the family very uneasy, thinking that he had the "winter fever." On examining him, I found the same state of affairs as in the last case, only more aggravated, the fever and pain lasting from noon until near midnight, but quinine arrested the fever, and in less than forty-eight hours he was running about town.

I have met with quite a number of similar cases, but these are enough to warrant the conclusion that unless the patient be carefully examined, it is easy to make out a case of pleurisy or pneumonia where none exists, and to cause the patient a week's sickness by depletion, when we are called on to treat a simple intermittent pleurodynia.

